# Influences of Maternal Conjugated Linoleic Acid and Essential Fatty Acid Supply During Late Pregnancy and Early Lactation on T and B Cell Subsets in Mesenteric Lymph Nodes and the Small Intestine of Neonatal Calves

**DOI:** 10.3389/fvets.2020.604452

**Published:** 2020-12-16

**Authors:** Wendy Liermann, Torsten Viergutz, Katrin Lena Uken, Laura Vogel, Martina Gnott, Dirk Dannenberger, Armin Tuchscherer, Hermine Kienberger, Michael Rychlik, Arnulf Tröscher, Harald Michael Hammon

**Affiliations:** ^1^Institute of Nutritional Physiology “Oskar Kellner”, Leibniz Institute for Farm Animal Biology (FBN), Dummerstorf, Germany; ^2^Institute of Reproductive Biology, Leibniz Institute for Farm Animal Biology (FBN), Dummerstorf, Germany; ^3^Institute of Muscle Biology and Growth, Leibniz Institute for Farm Animal Biology (FBN), Dummerstorf, Germany; ^4^Institute of Genetics and Biometry, Leibniz Institute for Farm Animal Biology (FBN), Dummerstorf, Germany; ^5^Bavarian Center for Biomolecular Mass Spectrometry, Freising, Germany; ^6^Analytical Food Chemistry, Technical University of Munich, Freising, Germany; ^7^BASF SE, Lampertheim, Germany

**Keywords:** calf, conjugated linoleic acids, essential fatty acids, T and B cell, small intestine, mesenteric lymph node, colostrum

## Abstract

Conjugated linoleic acid (CLA) isomers are known for their health-promoting effects in mammals and metabolic functions in dairy cows and are synthesized in the forestomach depending on essential fatty acid (EFA) intake. The current preliminary study investigated effects of a maternal fatty acid supplementation (MFAS) during late pregnancy and early lactation with coconut oil (CON, control), CLA (Lutalin®), or CLA + EFA (Lutalin® linseed oil; safflower oil) on plasma fatty acid composition and T and B cell subsets in mesenteric lymph nodes (MLN) and the small intestine of 5-day-old calves. MFAS of CLA + EFA increased α-linolenic, eicosapentaenoic, docosapentaenoic, and *n*-3 fatty acid proportions in calf plasma fat on days 1 and 5 after birth (*P* < 0.05). On day 5, CLA and CLA + EFA calves showed higher plasma fat *trans*-10, *cis*-12 CLA proportions, and CLA calves had higher plasma *cis*-9, *trans*-11 CLA proportions compared with CON calves (*P* < 0.1). MFAS of CLA tended to increase CD4^+^ T cell subsets in MLN and increased CD21^+^ B cell subsets in ileal lamina propria compared with CON but decreased CD2^+^ T cell subsets in jejunal lamina propria (*P* < 0.05). CLA + EFA decreased CD4^+^ T cell subsets in MLN compared with CLA (*P* < 0.05). MFAS of CLA seemed to affect the intestinal adaptive immune system of calves, but additional EFA supplementations reversed CLA effects. Possible direct CLA and EFA effects or whether changes in milk composition affected this immune modulation must be clarified in further studies.

## Introduction

Conjugated linoleic acid (CLA) isomers are known for their health-promoting effects in mammals and different metabolic functions in the dairy cow (1–3). In ruminants, they are mainly synthesized in the forestomach depending on the intake of essential fatty acids (EFAs), such as α-linolenic acid and linoleic acid (4, 5). One of the known functions of CLA supplementation in the dairy cow is its milk fat-reducing effect (3, 6, 7). In studies of Haubold et al. (3) and Vogel et al. (8), CLA influenced the chemical and fatty acid composition in colostrum and milk. Milk is the most important source of nutrients for the calf in the first weeks after birth. Therefore, these alterations might also have a direct impact on the calf because fatty acids and their metabolites are, besides their caloric effects, important cell membrane components, play a key role as substrates in many biochemical pathways, and are known as cell signaling molecules and immune modulators (9, 10). It is discussed that fatty acids and especially *n*-3 and *n*-6 fatty acids have crucial effects on T cell proliferation, cytokine production, and phenotypic differentiation (10–12). Also, derivates synthesized from polyunsaturated fatty acids such as eicosanoids seemed to take part in inflammation and regulation of T and B cell function (13). In turn, T cells may play a key role in the intestinal immune system (14). CLA seemed to have also local effects on intestinal health. Thus, it inhibited the onset of experimental inflammatory bowel disease in pigs (15). Further, maternal CLA supplementation reduced the intestinal mucosal inflammation in piglets after *Escherichia coli* infection (16).

With regard to the effects of CLA on the chemical and fatty acid composition in milk and its direct effects on the intestine, the present study aimed to investigate the effects of a maternal CLA supplementation during late pregnancy and early lactation either with or without EFA supplementation on the intestinal adaptive immune system in neonatal calves. To prove the transfer of CLA and EFA from the cow to the calves, the fatty acid composition in plasma of the neonatal calves was additionally determined.

## Materials and Methods

The present study was conducted at the experimental station of the Leibniz Institute for Farm Animal Biology, Dummerstorf. Housing conditions and experimental procedures were in accordance with the guidelines of the German Animal Protection Law and approved by the Landesamt für Landwirtschaft, Lebensmittelsicherheit und Fischerei Mecklenburg-Vorpommern, Rostock (registration number 7221.3-1-052/15).

### Experimental Design and Animals

Nine pregnant rumen-fistulated Holstein cows in second lactation were abomasally supplemented either with coconut oil (control, CON; 76 g/d; Bio-Kokosöl #665, Kräuterhaus Sanct Bernhard KG, Bad Ditzenbach, Germany) known as a saturated fatty acid source, 38 g/day Lutalin® (CLA, 10 g/day *cis*-9, *trans*-11, 10 g/day *trans*-10, *cis*-12 CLA; BASF SE, Ludwigshafen, Germany) or a combination of CLA and EFA [CLA + EFA; 38 g/day Lutalin®, 78 g/day linseed oil (DERBY® Leinöl #4026921003087, DERBY® Spezialfutter GmbH, Münster, Germany), and 4 g/day safflower oil (GEFRO® Distelöl, GEFRO® Reformversand Frommlet KG, Memmingen, Germany)] from 63 days before until day 5 after calving (8). During the dry period beginning 6 weeks before expected calving, each dose was halved. Cows were fed a corn silage-based total mixed ratio described by Vogel et al. (8).

The nine calves born from the supplemented cows (CON, *n* = 3, 2 male calves and 1 female calf; CLA, *n* = 3, 1 male calf and 2 female calves; CLA + EFA, *n* = 3, 2 male calves and 1 female calf) were separated from their dams immediately after birth and individually housed in boxes littered with straw and equipped with a water trough. The boxes (1.4 × 2.45 m) were integrated into a climate-controlled room with a constant temperature of 19°C. Calves were fed colostrum/transition milk from respective dams by nipple bottles. First colostrum was provided to calves within the first 3 h after birth. On day 1, colostrum was fed at an amount of 10% of body weight and, from day 3 on, at an amount of 12% of body weight, which was provided into two meals per day. On day 2, after birth, only 6% colostrum of body weight was fed, also provided into two meals to equal the amounts of provided colostrum within the first 48 h after birth between all calves. In general, the first colostrum was fed on day 1 of life, and on the following day's colostrum/transition milk gained in the morning of the respective day was fed. If these milkings were insufficient, the milk of the second milking of the respective day was used. If both milkings were insufficient, the colostrum/transition milk of a dam from the respective group and milking was added, which was conducted only in the case of one calf. Refused colostrum/transition milk was tube-fed.

### Sampling Procedures and Analyses

Milk samples were taken on day 1 (first colostrum) and day 5 after calving. Milk analyses according to dry matter content, crude protein, lactose, and crude fat content were done corresponding to methods described by Görs et al. (17). The fatty acid distribution in the first colostrum was quantified by lipid extraction and gas chromatography with flame ionization detection according to methods of Firl et al. (18).

Blood samples from the jugular vein of calves were collected in conventional Ethylenediaminetetraacetic acid tubes on day 1 of life before the first meal and on day 5 after birth before the morning feeding (0800 h). To select plasma from whole blood, samples were centrifuged at 2,700 × *g* and 4°C for 20 min. Plasma fatty acid composition was determined by gas chromatography according to methods described by Dannenberger et al. (19, 20).

The initial body weight of calves after birth and the final body weight on day 5 were determined before feeding. Furthermore, the body temperature was measured daily, in the morning and in the evening before feeding. On day 5 after birth, calves were slaughtered 2 h after feeding by bolt shooting and exsanguination. The whole gastrointestinal tract was dissected, and tissue samples from mid-jejunum, jejunal Peyer's patches (two to three Peyer's patches cranial to the mid-jejunum), terminal ileum (5 cm cranial of the ileocecal junction), and mesenteric lymph nodes (MLNs, close to the sample collection of mid-jejunum) were taken immediately after dissection within 15 min postmortem and stored in phosphate-buffered saline (PBS) on ice.

Leucocytes from MLN were isolated according to modified methods of Liermann et al. (21). Thus, surrounding fat tissue and serosa were removed, and thereafter, the MLN was transferred into a petri dish containing cold PBS (without Ca^2+^ and Mg^2+^; life technologies^TM^; Carlsbad, USA). The MLN was fragmented by a scalpel to release the cells. The tissue–cell mixture was transferred in MACS® SmartStrainers (mash size 70 μm; Miltenyi Biotec; Bergisch Gladbach, Germany), sieved, and collected in 50-ml tubes. The cell suspension was centrifuged at 300 × *g* for 10 min at room temperature. The supernatant was rejected, and the pellet was resuspended in 5-ml antibody buffer [4.9-ml PBS without Ca^2+^ and Mg^2+^, 10-mM 4-(2-hydroxyethyl)-1-piperazineethanesulfonic acid (Sigma Aldrich Chemie GmbH; Munich, Germany), 0.1-ml fetal bovine serum (Merck KGaA; Darmstadt, Germany), 2-mM ethylenediaminetetraacetic acid (life technologies^TM^)].

Jejunal and ileal intraepithelial lymphocytes were isolated by different washing and sieving steps and cells from lamina propria (LP) by enzymatic digestion. The cell isolation from both localizations was conducted simultaneously using the LP dissociation kit of Miltenyi Biotec following the manufacturer's protocol. The received cell suspensions were subsequently centrifuged at 300 × *g* for 10 min at room temperature. The supernatant was discarded, and the cell pellet was resuspended in antibody buffer as used for cells isolated from MLN.

The methods for the isolation of immune cells from Peyer's patches were based on mechanical disruption of the dissected tissue and different washing and sieving steps and were described in detail by Liermann et al. (21).

Cells were stained with monoclonal antibodies for CD2 (T cells) [mouse anti-bovine CD2: fluorescein isothiocyanate (FITC); Bio-Rad Laboratories, Hercules, USA], CD4 (T helper cells) (mouse anti-bovine CD4: FITC; Bio-Rad), CD21 (B cells) (mouse anti-bovine CD21: RPE, Bio-Rad), or the corresponding isotype controls (mouse IgG1 negative control: FITC; mouse IgG2a negative control: FITC; mouse IgG2b negative control: RPE). The cell suspension was incubated for 30 min at 4°C in the dark. After that, cells were washed by centrifugation at 250 × *g* for 5 min at 4°C and resuspended in 600-μl PBS in case of CD2 and CD21 staining or 1,000-μl PBS in case of CD4 staining. T and B cell subsets were quantified by flow cytometry (Gallios^TM^, Beckman Coulter GmbH, Krefeld Germany). At least 10,000 cells were counted. Cells were gated according to single cells and lymphocyte population according to the forward and side scatter, as demonstrated in Supplementary Figure 1. All nonspecific signals indicated by isotype controls were evaluated and compensated by the Kaluza Analysis software (Beckman Coulter GmbH).

### Statistical Analyses

Statistical analyses were conducted with SAS for Windows (SAS Institute Inc., Cary, USA, Version 9.4). Descriptive statistics and tests for normality were calculated with the UNIVARIATE procedure of Base SAS software. The data were approximately normally distributed and analyzed by analyses of variance using the MIXED procedure of SAS/STAT software. The analysis of variance model included the fixed factors maternal fatty acid supplementation (MFAS) and sex of calves. Least squares means (LSM) and corresponding standard errors were calculated for each fixed factor. All pair-wise differences between LSM were tested by the Tukey–Kramer procedure. Differences were declared as significant if *P* < 0.05. If there is no other statement, results are presented as LSM ± SE. A principal component (PC) analysis was done using JMP 13 to visualize relationships between 61 variables, including colostral and milk traits, fatty acid composition in first colostrum and plasma, and T and B cell subsets measured in the considered localizations and cases in a two-dimensional space based on correlations.

## Results

There were no significant differences regarding the body weight of calves from different feeding groups at birth or on slaughtering day (CON = 43.3 ± 1.2 kg; CLA = 40.6 ± 1.2 kg; CLA + EFA = 43.9 ± 1.2 kg). Although the birth weight did not differ between male (44.1 ± 1.3 kg) and female calves (38.7 ± 1.4 kg), the final body weight tended to be higher in male calves (46.8 ± 1.3 kg) compared with that in female calves (40.6 ± 1.4 kg) (*P* < 0.1). The body temperature did not differ among calves of different feeding groups or different sex and was within the physiological reference range in all calves (data not shown).

Colostral dry matter, crude protein, lactose, and fat content of first colostrum and milk on day 5 are summarized in Table 1, and fatty acid composition of first colostrum and in plasma fat of calves on days 1 and 5 are shown in Tables 1, 2.

**Table 1:** Dry matter content and chemical and fatty acid composition in milk depending on maternal fatty acid supplementation (MFAS^1^) (LSM ± SE, *n* = 9).

**Variable**	**MFAS**			**Sex**		***P*** **Values**	
	**CON (*n* = 3)**	**CLA (*n* = 3)**	**CLA+EFA (*n* = 3)**	**Male (*n* = 5)**	**Female (*n* = 4)**	**MFAS**	**Sex**
**First colostrum**							
Dry matter (DM), %	26.2 ± 1.4	22.9 ± 1.4	27.2 ± 1.4	25.4 ± 1.4	25.5 ± 1.6	0.17	0.96
CP, g/kg FM^2^	151 ± 18	121 ± 18	167 ± 18	132 ± 14	161 ± 15	0.27	0.23
CP, g/kg DM	576 ± 54	530 ± 54	615 ± 54	516 ± 42	631 ± 47	0.57	0.31
Lactose, g/kg FM	22.5 ± 1.7	22.8 ± 1.7	20.8 ± 1.7	21.7 ± 1.3	22.4 ± 1.5	0.71	0.72
Lactose, g/kg DM	86 ± 11	101 ± 11	78 ± 11	87 ± 8	90 ± 9	0.38	0.85
Fat, g/kg FM	56.7± 14.0	58.7 ± 14.0	49.8 ± 14.0	70.4 ± 10.8	39.7 ± 12.2	0.90	0.13
Fat, g/kg DM	216 ± 53	253 ± 53	181 ± 53	279 ± 41	154 ± 46	0.66	0.10
**Fatty acids, %^3^**							
Linoleic acid	2.96 ± 0.16^a^^,^ ^b^	2.40 ± 0.16^b^	3.36 ± 0.16^a^	3.06 ± 0.12	2.75 ± 0.14	0.024	0.17
α-linolenic acid	0.17 ± 0.15^b^	0.21 ± 0.15^b^	1.52 ± 0.15^a^	0.81 ± 0.12	0.46 ± 0.13	0.002	0.11
*cis*-9, *trans*-11 CLA	0.39 ± 0.07	0.35 ± 0.07	0.32 ± 0.07	0.38 ± 0.05	0.33 ± 0.06	0.59	0.55
*trans*-10, *cis*-12 CLA	0.28 ± 0.06	0.26 ± 0.06	0.27 ± 0.06	0.29 ± 0.05	0.24 ± 0.05	0.79	0.48
Arachidonic acid	0.25 ± 0.03	0.27 ± 0.03	0.30 ± 0.03	0.31 ± 0.03	0.23 ± 0.03	0.55	0.11
EPA^4^	0.04 ± 0.01^b^	0.03 ± 0.01^b^	0.15 ± 0.01^a^	0.09 ± 0.01	0.06 ± 0.01	0.003	0.20
DPA^5^	0.10 ± 0.03^b^	0.11 ± 0.03^b^	0.32 ± 0.03^a^	0.21 ± 0.03	0.14 ± 0.03	0.008	0.17
*n*-3^6^	0.30 ± 0.20^b^	0.25 ± 0.20^b^	1.99 ± 0.20^a^	1.10 ± 0.15	0.66 ± 0.17	0.003	0.12
*n*-6^7^	3.45 ± 0.19^a^^,^ ^b^	2.86 ± 0.19^b^	3.88 ± 0.19^a^	3.61 ± 0.15	3.19 ± 0.16	0.035	0.12
**Day 5 of life**							
Dry matter (DM), %	14.5 ± 0.8	14.1 ± 0.7	14.1 ± 0.7	14.0 ± 0.5	14.5 ± 0.6	0.87	0.57
CP, g/kg FM	39.4 ± 1.2	36.9 ± 1.2	40.2 ± 1.2	38.6 ± 0.9	39.0 ± 1.0	0.22	0.80
CP, g/kg DM	271 ± 10	263 ± 10	286 ± 10	277 ± 8	270 ± 9	0.37	0.61
Lactose, g/kg FM	37.5 ± 2.2	36.1 ± 2.2	37.2 ± 2.2	35.4 ± 1.7	38.8 ± 1.9	0.93	0.35
Lactose, g/kg DM	262 ± 23	258 ± 23	265 ± 23	254 ± 18	269 ± 20	0.99	0.69
Fat, g/kg FM	48.0 ± 6.6	45.3 ± 6.6	34.5 ± 6.6	41.1 ± 5.1	44.2 ± 5.7	0.36	0.71
Fat, g/kg DM	330 ± 32	318 ± 32	242 ± 32	290 ± 25	304 ± 28	0.20	0.73

1 *CON, control group = coconut oil. CLA, conjugated linoleic acid = Lutalin®. CLA + EFA = Lutalin® + linseed oil + safflower oil*.

2 *FM, fresh matter*.

3 *Proportions of presented fatty acids out of the total amount of fatty acids*.

4 *EPA, eicosapentaenoic acid*.

5 *DPA, docosapentaenoic acid*.

6 *Sum of n-3 fatty acids (18:3 cis-9, cis-12, cis-15; 18:4 cis-6, cis-9, cis-12, cis-15; 20:3 cis-11, cis-14, cis-17; 20:5 cis-5, cis-8, cis-11, cis-14, cis-17; 22:5 cis-7, cis-10, cis-13, cis-16, cis-19; 22:6 cis-4, cis-7, cis-10, cis-13, cis-16, cis-19)*.

7 *Sum of n-6 fatty acids (18:2 cis-9, cis-12; 18:3 cis-6, cis-9, cis-12; 20:2 cis-11, cis-14; 20:3 cis-8, cis-11, cis-14; 20:4 cis-5, cis-8, cis-11, cis-14; 22:2 cis-13, cis-16; 22:4 cis-7, cis-10, cis-13, cis-16; 22:5 cis-4, cis-7, cis-10, cis-13, cis-16)*.

a, b *Different lower case superscripts mark significant differences between feeding groups, and different capitalized superscripts designate significant differences between male and female calves (P < 0.05)*.

**Table 2:** Fatty acid composition in calf plasma depending on maternal fatty acid supplementation (MFAS^1^) (LSM ± SE, *n* = 9).

**Fatty acids^**2**^**	**MFAS**			**Sex**		***P*** **Values**	
	**CON (*n* = 3)**	**CLA (*n* = 3)**	**CLA+EFA (*n* = 3)**	**Male (*n* = 5)**	**Female (*n* = 4)**	**MFAS**	**Sex**
**Day 1 of life, %**							
Linoleic acid	4.50 ± 0.63	4.67 ± 0.63	5.06 ± 0.63	5.36 ± 0.49	4.12 ± 0.55	0.82	0.16
α-linolenic acid	0.09 ± 0.03^b^	0.13 ± 0.03^b^	0.41 ± 0.03^a^	0.24 ± 0.02	0.18 ± 0.03	0.001	0.14
*cis*-9, *trans*-11 CLA	0.05 ± 0.01	0.09 ± 0.01	0.09 ± 0.01	0.09 ± 0.01	0.06 ± 0.01	0.09	0.06
*trans*-10, *cis*-12 CLA	0.03 ± 0.02	0.04 ± 0.02	0.01 ± 0.02	0.05 ± 0.02	0.00 ± 0.02	0.63	0.15
Arachidonic acid	6.83 ± 0.83	8.03 ± 0.83	6.92 ± 0.83	8.39 ± 0.65	6.12 ± 0.73	0.58	0.07
EPA^3^	0.16 ± 0.18^b^	0.27 ± 0.18^b^	1.46 ± 0.18^a^	0.78 ± 0.14	0.46 ± 0.16	0.006	0.217
DPA^4^	1.49 ± 0.13	1.53 ± 0.13	1.94 ± 0.13	2.06 ± 0.10^A^	1.25 ± 0.12^B^	0.12	0.004
DHA^5^	1.03 ± 0.20^b^	0.99 ± 0.20^b^	2.05 ± 0.20^a^	1.53 ± 0.16	1.18 ± 0.18	0.022	0.21
*n*-3^6^	2.76 ± 0.43^b^	2.92 ± 0.43^b^	5.58 ± 0.43^a^	4.63 ± 0.34^A^	3.09 ± 0.38^B^	0.006	0.032
*n*-6^7^	15.2 ± 1.5	16.5 ± 1.5	16.1 ± 1.5	18.3 ± 1.2^A^	13.6 ± 1.3^B^	0.82	0.048
**Day 5 of life, %**							
Linoleic acid	24.5 ± 3.6	22.8 ± 3.6	24.7 ± 3.6	24.8 ± 2.8	23.2 ± 3.1	0.92	0.72
α-linolenic acid	1.44 ± 0.27^b^	1.32 ± 0.27^b^	7.65 ± 0.27^a^	3.44 ± 0.21	3.50 ± 0.24	<0.001	0.87
*cis*-9, *trans*-11 CLA	0.23 ± 0.02^b^	0.37 ± 0.02^a^	0.30 ± 0.02^a, b^	0.30 ± 0.02	0.31 ± 0.02	0.017	0.7
*trans*-10, *cis*-12 CLA	0.00 ± 0.02^b^	0.16 ± 0.02^a^	0.18 ± 0.02^a^	0.12 ± 0.02	0.10 ± 0.02	0.005	0.41
Arachidonic acid	4.33 ± 0.42	3.66 ± 0.42	3.35 ± 0.42	3.71 ± 0.33	3.86 ± 0.37	0.23	0.78
EPA	0.48 ± 0.06^b^	0.36 ± 0.06^b^	1.50 ± 0.06^a^	0.73 ± 0.05	0.83 ± 0.05	<0.001	0.23
DPA	0.52 ± 0.06^b^	0.46 ± 0.06^b^	0.96 ± 0.06^a^	0.63 ± 0.05	0.66 ± 0.05	0.004	0.68
DHA	0.36 ± 0.05^a, b^	0.27 ± 0.05^b^	0.58 ± 0.05^a^	0.37 ± 0.04	0.44 ± 0.04	0.016	0.31
*n*-3	2.9 ± 0.3^b^	2.5 ± 0.3^b^	10.8 ± 0.3^a^	5.3 ± 0.3	5.5 ± 0.3	<0.001	0.57
*n*-6	30.8 ± 4.0	28.2 ± 4.0	29.6 ± 4.0	30.2 ± 3.1	28.8 ± 3.5	0.9	0.7

1 *CON, control group = coconut oil. CLA, conjugated linoleic acid = Lutalin®. CLA + EFA = Lutalin® + linseed oil + safflower oil*.

2 *Proportions of presented fatty acids out of the total amount of fatty acids*.

3 *EPA, eicosapentaenoic acid*.

4 *DPA, docosapentaenoic acid*.

5 *DHA, docosahexaenoic acid*.

6 *Sum of n-3 fatty acids (18:3 cis-9, cis-12, cis-15; 18:4 cis-6, cis-9, cis-12, cis-15; 20:3 cis-11, cis-14, cis-17; 20:5 cis-5, cis-8, cis-11, cis-14, cis-17; 22:5 cis-7, cis-10, cis-13, cis-16, cis-19; 22:6 cis-4, cis-7, cis-10, cis-13, cis-16, cis-19)*.

7 *Sum of n-6 fatty acids (18:2 cis-9, cis-12; 18:3 cis-6, cis-9, cis-12; 20:2 cis-11, cis-14; 20:3 cis-8, cis-11, cis-14; 20:4 cis-5, cis-8, cis-11, cis-14; 22:2 cis-13, cis-16; 22:4 cis-7, cis-10, cis-13, cis-16; 22:5 cis-4, cis-7, cis-10, cis-13, cis-16)*.

a,b;A,B *Different lower case superscripts mark significant differences between feeding groups, and different capitalized superscripts designate significant differences between male and female calves (P < 0.05)*.

The CLA + EFA supplementation resulted in a significant increase of linoleic acid and *n*-6 fatty acid proportions in the first colostrum compared with the CLA group (*P* < 0.05) and increased α-linolenic acid, eicosapentaenoic acid, docosapentaenoic acid, and *n*-3 fatty acid proportions compared with the CON and CLA group (*P* < 0.05). Similar effects were shown in the plasma of calves on days 1 and 5 of life (*P* < 0.05) except for linoleic acid, docosapentaenoic acid, and *n*-6 fatty acid proportion. Docosapentaenoic acid was only affected on day 5 of life, and linoleic acid and *n*-6 fatty acid proportions in calf were not affected in calf plasma (*P* > 0.05). Additionally, CLA + EFA calves showed higher proportions of docosahexaenoic acid in plasma compared with the other groups on days 1 and 5 of life (*P* < 0.05).

On day 5 of life, CLA calves showed higher *cis*-9, *trans*-11 CLA in plasma compared with CON calves (*P* < 0.015). Additionally, CLA and CLA + EFA calves had higher plasma *trans*-10, *cis*-12 CLA proportions compared with the control group on this day (*P* = 0.052).

CLA tended to increase the CD4^+^ T cell subsets in MLN (*P* = 0.068) and increased the CD21^+^ B cell subsets in the ileal LP (*P* = 0.020) but decreased the CD2^+^ T cell subsets in the jejunal LP compared with the CON group (*P* = 0.023) (Table 3). The MFAS with CLA + EFA decreased the CD4^+^ T cell subsets in MLN of calves compared with the CLA group (*P* < 0.05).

**Table 3:** T and B cell subsets in different localizations in 5-day-old calves depending on maternal fatty acid supplementation (MFAS^1^) (LSM ± SE, *n* = 9).

**Cells, %^3^**	**MFAS**			**Sex**		***P*** **Values**	
	**CON (*n* = 3)**	**CLA (*n* = 3)**	**CLA + EFA (*n* = 3)**	**Male (*n* = 5)**	**Female (*n* = 4)**	**MFAS**	**Sex**
**Mesenteric lymph nodes**							
CD2^+^ T cells	59.5 ± 9.4	42.5 ± 9.4	22.4 ± 9.4	41.1 ± 7.3	41.8 ± 8.2	0.09	0.95
CD4^+^ T cells	37.6 ± 4.9^a, b^^,^ ^2^	53.7 ± 4.1^a^	17.2 ± 4.1^b^	46.0 ± 3.5^A^^,^ ^2^	26.3 ± 3.5^B^	0.009	0.018
CD21^+^ B cells	31.1 ± 8.6	15.2 ± 8.6	42.2 ± 8.6	27.0 ± 6.7	32.0 ± 7.5	0.19	0.65
**Intraepithelial cells of jejunum**							
CD2^+^ T cells	71.7 ± 11.1	38.6 ± 11.1	56.7 ± 11.1	53.7 ± 8.6	57.7 ± 9.7	0.21	0.78
CD4^+^ T cells	10.4 ±4.5^2^	15.7 ± 3.7	3.5 ± 3.7	16.6 ± 3.3^A^^,^ ^2^	3.1 ± 3.3^B^	0.19	0.045
CD21^+^ B cells	0.4 ± 10.5	26.5 ± 10.5	0.6 ± 10.5	8.5 ± 8.2	9.8 ± 9.2	0.24	0.92
**Jejunal lamina propria**							
CD2^+^ T cells	49.1 ± 7.1^a^	7.8 ± 7.1^b^	40.4 ± 7.1^a, b^	12.5 ± 5.5^B^	52.3 ± 6.2^A^	0.023	0.006
CD4^+^ T cells	11.2 ± 5.2^2^	17.6 ± 4.3	8.3 ± 4.3	18.6 ± 3.8^2^	6.2 ± 3.8	0.4	0.08
CD21^+^ B cells	0.0 ± 12.1	28.3 ± 12.1	0.0 ± 12.1	19.7 ± 9.4	0.0 ± 10.6	0.22	0.15
**Jejunal peyer's patches**							
CD2^+^ T cells	18.0 ± 11.0	47.1 ± 11.0	13.6 ± 11.0	30.8 ± 8.6	21.7 ± 9.6	0.17	0.52
CD4^+^ T cells	19.6 ± 12.1^2^	34.5 ± 10.1	6.8 ± 10.1	24.7 ± 12.2^2^	16.1 ± 12.2	0.28	0.58
CD21^+^ B cells	10.8 ± 12.3	24.4 ± 12.3	49.8 ± 12.3	26.0 ± 9.6	30.7 ± 10.7	0.16	0.76
**Intraepithelial cells of ileum**							
CD2^+^ T cells	29.4 ± 6.9	18.5 ± 6.9	34.1 ± 6.9	24.3 ± 5.3	30.5 ± 6.0	0.25	0.49
CD4^+^ T cells	10.2 ± 6.8^2^	12.4 ± 5.7	21.4 ± 5.7	21.6 ± 5.0^2^	7.8 ± 5.0	0.45	0.12
CD21^+^ B cells	53.5 ± 12.6	27.8 ± 12.6	17.8 ± 12.6	33.0 ± 9.8	33.0 ± 11.1	0.21	1
**Ileal lamina propria**							
CD2^+^ T cells	2.1 ± 0.9	1.2 ± 0.9	1.8 ± 0.9	1.0 ± 0.7	2.4 ± 0.8	0.82	0.25
CD4^+^ T cells	8.1 ± 3.8^2^	14.1 ± 3.2	12.6 ± 3.2	12.7 ± 2.8^2^	10.6 ± 2.8	0.52	0.63
CD21^+^ B cells	0.0 ± 12.2^a^	73.2 ± 12.2^b^	18.0 ± 12.2^a, b^	35.1 ± 9.5	25.3 ± 10.7	0.021	0.53

1 *CON, control group = coconut oil. CLA, conjugated linoleic acid = Lutalin®. CLA + EFA = Lutalin® + linseed oil + safflower oil*.

2 *One animal has to be excluded because of technical difficulties during flow cytometry*.

3 *Cells in % of gated cells*.

a, b;A, B *Different lower case superscripts mark significant differences between feeding groups, and different capitalized superscripts designate significant differences between male and female calves (P < 0.05)*.

**Figure 1:**
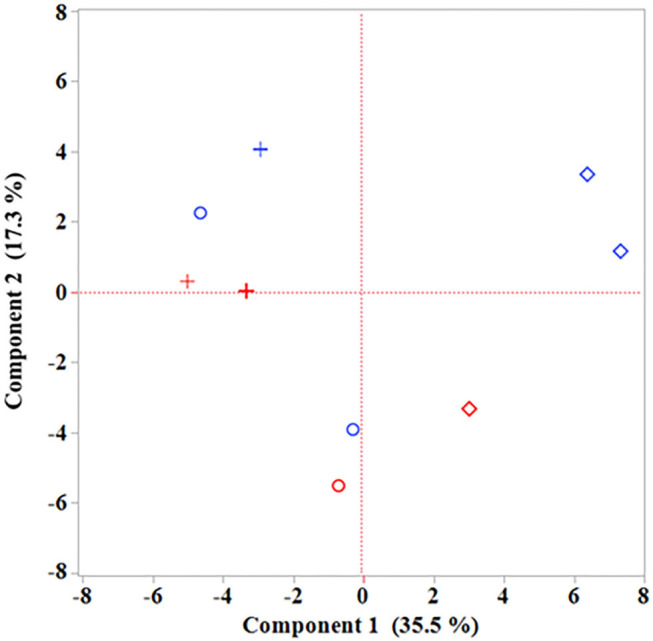
Projection of cases (calves; blue, male calves; red, female calves; °, control group; +, CLA group; ♢, CLA + EFA group) of the Principal component (PC) analysis for visualization of the relationships between 61 selected variables: dry matter content in colostrum (day 1 after calving) and transition milk (day 5 after calving); crude protein, fat, and lactose content in colostrum and transition milk on dry matter basis and fresh matter basis, linoleic acid, α-linolenic acid, arachidonic acid, *cis*-9, *trans*-11 CLA, *trans*-10, *cis*-12 CLA, eicosapentaenoic acid, docosapentaenoic acid, docosahexaenoic acid, *n*-3 fatty acid, and *n*-6 fatty acid proportions in first colostrum and calf plasma on days 1 and 5 of life; CD2^+^ T cell subsets, CD4^+^ T cell subsets and CD21^+^ B cell subsets in mesenteric lymph nodes, jejunal epithelium, jejunal lamina propria, ileal epithelium, ileal lamina propria, and jejunal Peyer's patches.

A PC analysis was performed to visualize relationships between 61 variables belonging to macronutrients in colostrum and milk, the fatty acid composition in the first colostrum and plasma of calves, and T and B cell subsets measured in MLN and the intestine (Figure 1 and Supplementary Figure 2). The analysis revealed the first two PC, which extracted ~52.8% of the total variance. According to a scree plot and the eigenvalue, consecutive PCs were assessed. The mean eigenvalue of 1.0 corresponded to a total of eight extracted components, which explained cumulatively 100% of the total variance between the 61 variables. With regard to the protection of cases, it became clear that CLA and CLA + EFA calves differed markedly from each other in the selected variables. Although calves from the CLA group seemed to correlate negatively to PC1, calves from the CLA + EFA group seemed to correlate positively to PC1.

## Discussion

The milk fat-reducing effect of CLA appeared to be offset in the colostral period. In the studies of Hötger et al. (7) and Vogel et al. (8), the CLA effect on milk fat depends on the time relative to calving and is less pronounced in the first week after calving.

Although only marginal proportions of CLA isomers were detected in the plasma of calves on day 1 of life, the increase of CLA from day 1 to 5 of life indicates an active transfer of the CLA by milk and also an increase of CLA in the milk of dams receiving an additional CLA supplementation, which might be supported by observations of Vogel et al. (8).

Because of the already existing differences in plasma fatty acid composition of CLA + EFA calves compared with calves of the other two groups immediately after birth, a partial placental transfer of EFA is suggested. A maternal transfer of supplemented fatty acids by the placenta was also indicated in previous studies of Noble et al. (22) and Garcia et al. (23). The differences became more pronounced on day 5 after birth, which might be based on the differences in milk fatty acid composition evoked by the MFAS. The fatty acid composition was only measured in the first colostrum in the present study, but in studies of Vogel et al. (8), it was demonstrated that the fatty acid supplementation resulted in continuously elevated *n*-3 fatty acid concentrations in milk during late and early lactation.

The present results indicated an effect of CLA on the CD2^+^ T cell subsets of jejunal LP and CD21^+^ B cell subsets of the ileal LP and also a trend on CD4^+^ T cells subsets in MLN, but an additional maternal supplementation of EFA seemed to reverse the effects of CLA. The shift in CD2^+^ T cell subsets in CLA might be a result of numerically increasing CD21^+^ B cell subsets in the jejunal LP. The numerical increase of CD21^+^ B cell subsets in the jejunal LP and the marked increase of CD21^+^ B cell subsets in the ileal LP of CLA calves could be based on the numerically higher CD4^+^ T cell subset because T helper cells can activate the B cell proliferation (24). In many cases, activated B cells will be differentiated into plasma cells producing secretory IgA, which plays a key role in the protection against infections (25). For concrete analyses in further studies, the cytokine and also the IgA production have to be considered. In the case of MLN, the higher CD4^+^ T cell subsets did not show simultaneously increased B cell subsets.

The possibility that T and B cell subsets can be affected by different MFAS was also demonstrated in studies of Binter et al. (26), testing piglets born and suckled by sows fed sunflower oil or seal blubber oil. Similar to the present study, these authors described effects of MFAS on the T and B cell subsets in the LP and MLN but not in lymphocyte populations of the intestinal epithelium, which might be due to the fast pace of changes in the epithelium. In general, it is suggested that detected immune modulations were based on local effects in the jejunum and ileum, but effects on immune cells in storage organs such as MLN might also indicate systemic effects. However, it was shown that the MFAS also influences the fatty acid composition in the MLN (26). Therefore, a local effect cannot be ruled out. Because of existing differences in fatty acid composition in plasma on day 1 after birth, an *in utero* priming effect of the fetal immune system by placental transfer of fatty acids might also be possible.

Indeed, it is thinkable that the presence of MFAS in CLA and CLA + EFA calves might be responsible for the local immune modulations because, in general, fatty acids modulate the proliferation of T cells in low concentrations, but high concentrations of longer and unsaturated fatty acids can also induce apoptosis (10). Further, it was shown that several fatty acids could modulate the cytokine production and release of T cells (10). The *n*-3 and *n*-6 fatty acids especially influence the cytokine production of T helper cells and, therefore, their phenotypic differentiation and subsequently their pro- or anti-inflammatory response (10–12). Studies by different authors demonstrated that T cells are directly able to recognize various types of fatty acids by fatty acid receptors (10, 27). Because of the active transfer of CLA by the milk as indicated by calf plasma concentrations also, a direct impact of the CLA isomers on the local immune modulations might be possible.

Interestingly, in studies of Bassaganya-Riera and Hontecillas (15), CLA inhibited the onset of experimentally induced inflammatory bowel disease in the porcine colon, but in combination with *n*-3 polyunsaturated fatty acids, the beneficial effects of CLA were offset. The higher *n*-3 fatty acid proportions in colostrum and plasma of EFA calves indicated a higher *n*-3 polyunsaturated fatty acids transfer to the calves in the current study. Possibly, these fatty acids might have also inhibited the effects of CLA in calves received milk from CLA + EFA dams.

The PC analysis underlined the overall differences between calves of the CLA and CLA + EFA group in plasma fatty acid composition and the T and B cell subsets and revealed relations to differences in milk composition.

The biological relevance of the present findings has to be studied during immune challenges and functional studies of T and B cells isolated from the bovine intestine and with a higher number of animals.

According to the present study, it can be concluded that the MFAS of CLA affects the local intestinal immune system of neonatal calves. However, an additional EFA supplementation reversed effects demonstrated in calves from CLA supplemented dams. In further studies considering more animals, the relationships between changes in colostral and milk composition and the adaptive intestinal immune system should be investigated in more detail. In one of the next steps, the biological role of the observed changes associated with the intestinal immune system has to be evaluated with respect to the local and systemic immune response in neonatal calves.

## Data Availability Statement

The raw data supporting the conclusions of this article will be made available by the authors, without undue reservation.

## Ethics Statement

The animal study was reviewed and approved by Landesamt für Landwirtschaft, Lebensmittelsicherheit und Fischerei Mecklenburg-Vorpommern, Rostock.

## Author Contributions

WL and HH: conceptualization. WL, TV, DD, HK, MR, and ATu: methodology. WL, KU, and LV: investigation. ATr and HH: resources and funding acquisition. WL: writing-original draft preparation. TV and HH: writing-review and editing. HH: supervision and project administration. All authors contributed to the article and approved the submitted version.

## Conflict of Interest

The authors declare that the research was conducted in the absence of any commercial or financial relationships that could be construed as a potential conflict of interest.

## Abbreviations

CLA: conjugated linoleic acid
CON: control
EFA: essential fatty acids
LP: lamina propria
LSM: least squares means
MFAS: maternal fatty acid supplementation
MLN: mesenteric lymph nodes
PC: principal component.
