# Supplementary figures and images for: Influences of Maternal Conjugated Linoleic Acid and Essential Fatty Acid Supply During Late Pregnancy and Early Lactation on T and B Cell Subsets in Mesenteric Lymph Nodes and the Small Intestine of Neonatal Calves
Source: Front Vet Sci. 2020 Dec 16;7:604452. doi: 10.3389/fvets.2020.604452 (PMC7772138; doi:10.3389/fvets.2020.604452)

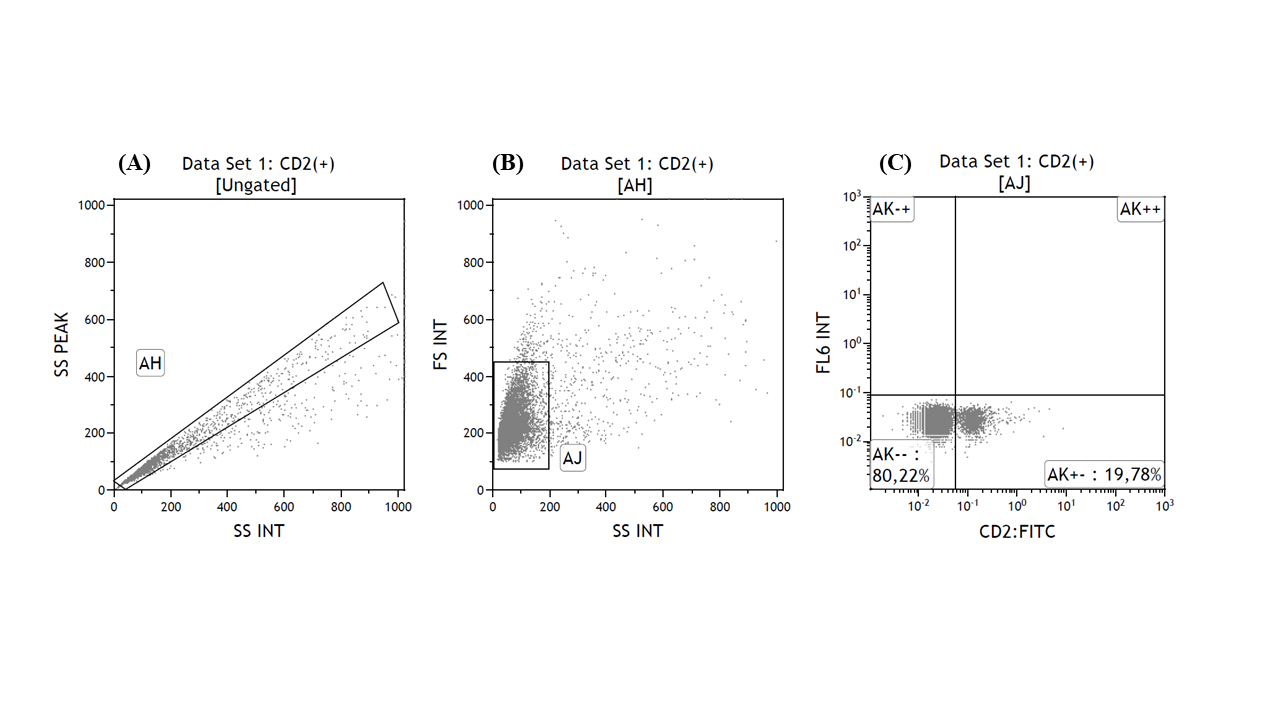

Supplement: Supplementary file 1 [file Image_1.TIF]

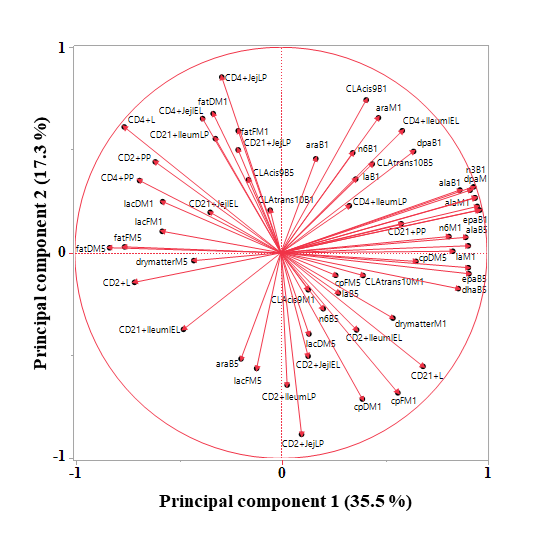

Supplement: Supplementary file 2 [file Image_2.TIF]
